# The Syntax and Meaning of Wild Gibbon Songs

**DOI:** 10.1371/journal.pone.0000073

**Published:** 2006-12-20

**Authors:** Esther Clarke, Ulrich H. Reichard, Klaus Zuberbühler

**Affiliations:** 1 School of Psychology, University of St Andrews, St Andrews, Scotland; 2 Max Planck Institute for Evolutionary Anthropology, Leipzig, Germany; 3 Department of Anthropology, Southern-Illinois University, Carbondale, United States of America; University of Cambridge, United Kingdom

## Abstract

Spoken language is a result of the human capacity to assemble simple vocal units into more complex utterances, the basic carriers of semantic information. Not much is known about the evolutionary origins of this behaviour. The vocal abilities of non-human primates are relatively unimpressive in comparison, with gibbon songs being a rare exception. These apes assemble a repertoire of call notes into elaborate songs, which function to repel conspecific intruders, advertise pair bonds, and attract mates. We conducted a series of field experiments with white-handed gibbons at Khao Yai National Park, Thailand, which showed that this ape species uses songs also to protect themselves against predation. We compared the acoustic structure of predatory-induced songs with regular songs that were given as part of their daily routine. Predator-induced songs were identical to normal songs in the call note repertoire, but we found consistent differences in how the notes were assembled into songs. The responses of out-of-sight receivers demonstrated that these syntactic differences were meaningful to conspecifics. Our study provides the first evidence of referential signalling in a free-ranging ape species, based on a communication system that utilises combinatorial rules.

## Introduction

Primates typically produce acoustic signals when detecting a predator, such as a raptor, large cat, or snake. These vocalisations are termed alarm calls, which function to warn other group members and sometimes also to communicate directly to the predator, for example to attract its attention or advertise perception [1]. For some primates, there is good evidence for reference-like communication. If signallers reliably produce acoustically distinct vocalisations to different classes of predators, then recipients often respond to them as if they have spotted the corresponding predator themselves, presumably because they draw inferences about ongoing external events from the calls [2]. Very little is known about the psychological processes that underlie and drive call production, and hence the term ‘functionally referential’ is sometimes used to describe such cases. In contrast to human speech, such communication systems are not necessarily based on signallers and recipients understanding each other's intentions [3]. Instead, the default assumption is that primates and other animals do not vocalise to actively inform each other, but information is transmitted as a by-product of signallers responding to evolutionarily important events.

Somewhat strikingly, there is relatively little evidence for referential signalling from our closest living relatives, the apes. This is particularly puzzling in the light of a substantial literature on referential signalling in various monkey species, such as vervet monkeys, Diana monkeys, Campbell's monkeys, putty-nosed monkeys, or white-faced capuchin monkeys [4]–[8]. Recently, it has been suggested that captive chimpanzees produce functionally referential calls in response to different types of foods, but it is not yet clear what features of the food the calls denote [9]–[10].

Gibbons (*Hylobatidae*), the South-East Asian forests' smaller apes, are known for a most remarkable behaviour, their loud and conspicuous songs that transmit over long distances, often up to one kilometre or more through dense forest vegetation [11]. Although mostly monogamous, gibbons occasionally engage in extra-pair copulations and take-overs of residents by males or females have been documented [12]–[13]. In most gibbon species, the mated pair normally sings in the morning in a coordinated fashion, the so-called duet song, most likely to communicate to neighbouring individuals [14]–[19]. Gibbons sometimes also produce songs in response to predators, but no systematic study has ever been conducted to investigate this phenomenon [20]–[21]. Their body mass is small compared to other members of the Hominoidea, more comparable to that of a large monkey (5 to 11 kg). This is likely to make them vulnerable to predation from large cats, snakes and birds of prey and, perhaps because of this, gibbons almost exclusively reside in the upper forest canopy [22]–[25]. We were interested to what extent white-handed gibbons of Khao Yai National Park, Thailand, produced songs in response to potential predators and whether these songs differed from songs given as part of the daily morning routine, the duets.

## Methods

### Study site and subjects

Khao Yai National Park is situated approximately 130 km NE of Bangkok, Thailand (101°22′E, 14°26′N). Data were collected at the Central Mo Singto study site, situated at an elevation of 730–860 m [26]. Habituation of groups started during the 1980ies. At the time of the study, 13 groups were available for observations with all group members individually known and social histories documented for most of them (Table 1).

**Table 1:**
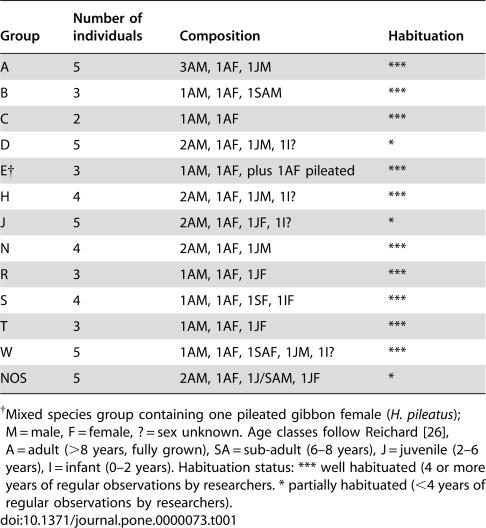
Composition of study groups (August 2005)

Group	Number of individuals	Composition	Habituation
A	5	3AM, 1AF, 1JM	***
B	3	1AM, 1AF, 1SAM	***
C	2	1AM, 1AF	***
D	5	2AM, 1AF, 1JM, 1I?	*
E†	3	1AM, 1AF, plus 1AF pileated	***
H	4	2AM, 1AF, 1JM, 1I?	***
J	5	2AM, 1AF, 1JF, 1I?	*
N	4	2AM, 1AF, 1JM	***
R	3	1AM, 1AF, 1JF	***
S	4	1AM, 1AF, 1SF, 1IF	***
T	3	1AM, 1AF, 1JF	***
W	5	1AM, 1AF, 1SAF, 1JM, 1I?	***
NOS	5	2AM, 1AF, 1J/SAM, 1JF	*

† Mixed species group containing one pileated gibbon female (*H. pileatus*); M = male, F = female, ? = sex unknown. Age classes follow Reichard [26], A = adult (>8 years, fully grown), SA = sub-adult (6–8 years), J = juvenile (2–6 years), I = infant (0–2 years). Habituation status: *** well habituated (4 or more years of regular observations by researchers. * partially habituated (<4 years of regular observations by researchers).

Study groups consisted of between 2 to 6 individuals, mostly an adult pair and their offspring, sometimes with more than one adult male [27]–[29]. Males and females disperse from their natal group at or some years after reaching sexual maturity, starting at about 7–8 years of age. White-handed gibbons are sexually monomorphic, and of either light or dark pelage colour, although this is unrelated to sex or age. The Central Mo Singto study site borders on the distribution area of the pileated gibbon (*Hylobates pileatus*), and a hybrid zone exists between the two species.

### Vocal behaviour

**Figure 1:**
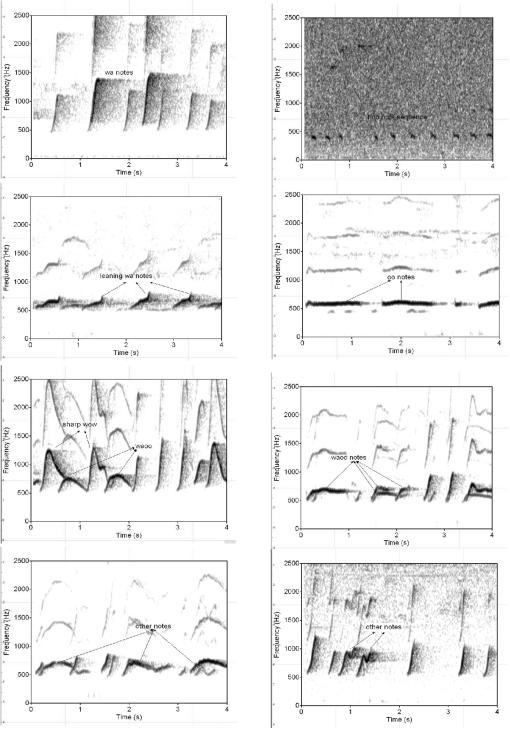
Gibbon song notes as distinguished by Raemaekers et al. [**18**] **:** (1) The ‘wa’ note is a short and steeply rising note, appearing as a more or less straight line on the spectrogram; sometimes appearing slightly concave. It consistently spans over 100 Hz in the frequency domain, which sets it aside from the ‘hoo’ note. (2) The ‘hoo’ is a low frequency quiet note consistently spanning a much narrower frequency range than ‘wa’ notes. (3) The ‘leaning wa’ notes may be more or less straight like the ‘wa’ notes but longer in duration, and therefore lean more to the right; sometimes they have a slight bump in the middle. (4) The ‘oo’ note is of a relatively even pitch and therefore produces a flat note, as seen on the spectrogram, of varying duration. Sometimes it may rise slightly at the start. (5) The ‘sharp wow’ note is a loud and penetrating note. It rises steeply at first then falls steeply to produce a concave curve. It invariably spans more than 700 Hz in the frequency domain. The end of the note may be prolonged horizontally. (6) The ‘waoo’ note is highly variable. It always rises steeply at first, but then may hold pitch at an even level or fall in pitch to create a convex curve. It spans a much lower frequency range than the ‘sharp wow’. (7) Notes that did not fit in with the shapes and definitions of the other six notes described above were allocated as ‘other’. These were highly variable, and some may warrant their own unique note category, but for the purposes of this study they are grouped together. This category also describes the above six note shapes when given with major pitch modulations that give them a wobbly or trembling quality. Finally, the ‘ooaa’ is extremely rare and was not found in any of the analysed recordings in this study, and so is not described here.

**Figure 2:**
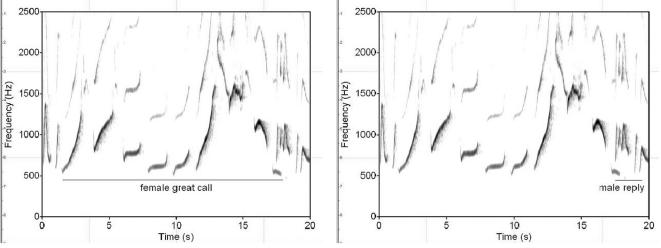
The female great call and male reply phrases. The female great call is a loud and penetrating two-humped call that is largely invariable within and between individuals, lasting on average 17.4 seconds (±1.32, n = 13, duets and predator contexts). The male reply is similarly stereotyped and usually follows the female call swiftly (underlined portion).

(a) **Single notes:** The basic vocal behaviour of white-handed gibbons has been described already [18] and, whenever possible, we used the same terminology (fig. 1). Individual vocal units are termed ‘notes’, of which seven different types can be distinguished: (1) ‘wa’, (2) ‘hoo’, (3) ‘leaning wa’, (4) ‘oo’, (5) ‘sharp wow’, (6) ‘waoo’, and (7) ‘other’. The ‘hoo’ was originally considered part of the ‘wa’ group, but we found that this class was consistently lower-pitched than ‘wa’ (mean frequency = 73.8 Hz±36.9, n = 115), of lower amplitude, and usually covering a frequency range of less than 100 Hz. These three parameters were perceptually salient and they allowed us to reliably discriminate between ‘wa’ and ‘hoo’ notes.

(b) **Note combinations:** Individual notes are rarely produced in isolation but are normally assembled into more complex structures, so-called ‘figures’ or ‘phrases’, to form a ‘song’. Figure 2 depicts two prominent examples of phrases, the relatively rigid ‘female great call’ and the ‘male reply’. If two group members produce songs in a coordinated way, this is termed a ‘duet’.

### Data collection and equipment

The study was undertaken between April 2004 and August 2005. All calls were recorded using Sony DAT recorders (TCD-D8 and TCD-D7), and Sennheiser directional microphones (MKH815T and ME66) with windshields. *Ad libitum* recordings throughout the day resulted in a library of natural duet songs (n = 14) from different groups, which we then compared with predator-induced songs. Natural predator encounters are very difficult to observe in dense rainforest habitats and systematic studies are almost impossible to conduct. Hence, we elicited songs by experimentally presenting realistic life-size predator models to the different study groups.

**Figure 3:**
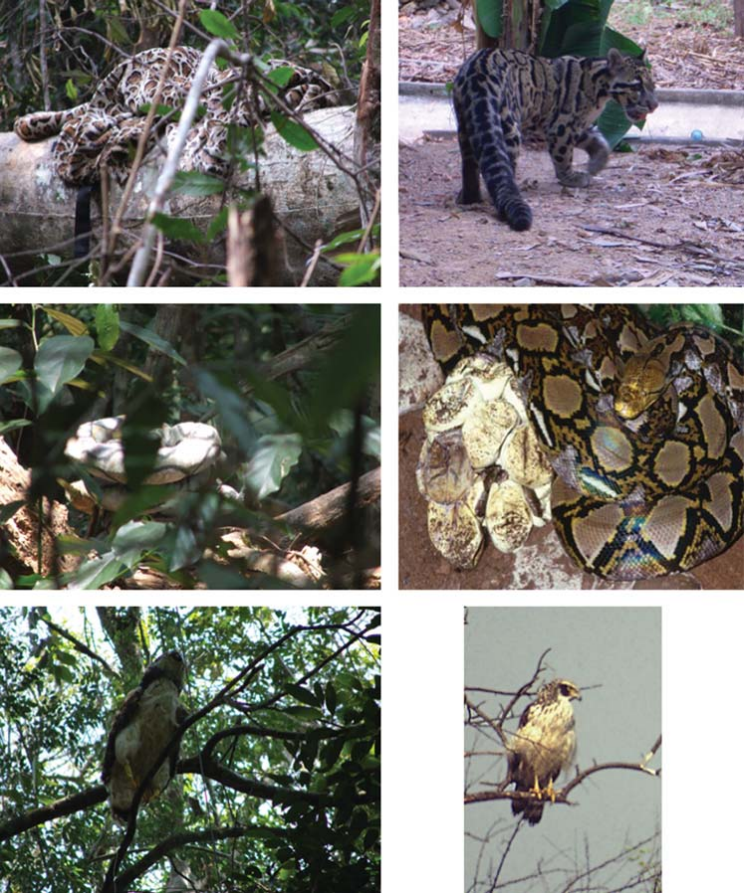
**Predator models used in the experiments:** (a) leopard model compared to a real clouded leopard, (b) python model compared to a real reticulated python, (c) eagle model compared to a real crested serpent eagle (photographs by E.C, Brendon Snyder, Anna Wilkinson, Jörg Hess, and Liz Leyden, printed with the authors' permissions).

Our model predators were custom-made, to match photographs of the real predators, and positioned in their natural resting or hiding position [30] (see fig. 3). We tested the gibbons' responses to the following predator types: clouded leopard (*Neofelis nebulosa*), tiger (*Panthera tigris*), reticulated python (*Python reticulates*), and crested serpent eagle (*Spilornis cheela*). For the tiger and clouded leopard models, we used two different exemplars. Clouded leopard models consisted of fake-fur wrapped around an object, such as a large rucksack, positioned approximately 1 m from the ground on a log or stone. Tiger models consisted of a person covered by fake-fur. The reticulated python model was made from several draught excluders of approximately 10 cm in diameter, sewn together and painted, to make a tube of about 4 metres in length. The python was placed, usually coiled, at about 1–2 metres above the forest floor in a small tree, tree stump, or log. The crested serpent eagle model, finally, was made from chicken wire and papier-mâché, painted and feathered with feather dusters bought locally. It was hoisted 4–10 m into the trees with a rope, and positioned usually between two parallel branches as though perched on the lower branch. For this, we shot a weight attached to a fishing line over an appropriate branch with a catapult, allowing us to pull the rope over the branch for hosting and positioning the eagle model into the canopy.

Our overall aim was to keep stress for the study animals as low as possible. Some of our observations suggested that natural predation events could occur at a maximum rate of about one per 3–4 days. We decided to present predator models at intervals of no more than one per week per group, considerably below the maximum observed rate. Each group was exposed only once, maximally twice, with a particular model type. Predator models were presented in open forest habitat, so that individuals always had open escape routes.

### Experimental protocol

Groups were located usually by their morning duets, or by identifying their sleeping site the night before. Once found, the observer (EC) followed them for at least 2 hours before an experimental trial was initiated. This period permitted the individuals to habituate to the observer's presence and it provided baseline vocal and non-vocal data before model presentation. If no real predator was encountered during this 2-hour period, a predator model was positioned so that the subjects could not observe the procedure. The model was then displayed for a period of about 20 minutes total, starting from when the gibbons had detected it, and then removed. The observer usually remained with the group for at least two more hours, or until the group reached their final sleeping site for the day. Throughout model presentation, the focal individuals' behaviour was monitored continuously and recordings were made of their calls. The vocal responses of neighbouring groups were also recorded whenever they occurred.

### Data analyses

As outlined, we were interested in the structural differences of gibbon songs produced (a) as part of their early morning routine and (b) in response to predators. We decided to exclude the responses to the tiger model from the main analyses because of the rarity of real tigers in the study site. Since January 1999, only two sightings have been made in the entire park, despite intensive sampling efforts [31]. Nevertheless, groups responded strongly to this predator model, in one case also triggering a vocal response from a neighbouring group.

Whenever we recorded neighbour responses to the focal groups' singing behaviour to a predator, we analysed these calls as well, as this provided us with a natural experiment: Since we knew exactly what the focal group responded to, we could determine what information their songs potentially transmitted to recipients in adjacent home-ranges or to group members who were temporarily away from the group. We included the response to the tiger model for this analysis due to the low sample size.

Calls were digitised using Cool Edit 2000 software. Spectrograms were made using Raven 1.2.1 with a Hanning window function, 8.71 Hz filter bandwidth, 0.5 Hz frequency resolution and 15 s grid time resolution. Gibbons' singing is a crescendo of notes, particularly in response to predators. Vocal behaviour usually starts with a series of very soft ‘hoo’ notes, initially only audible at close range, but rapidly grading into much louder units carrying over long distances. Hence, for each song we defined its start as the first loud non-‘hoo’ note. Then, we determined the following: (a) number of ‘hoo’ notes and (b) duration of ‘hoo’ sequence before song onset. After song onset we determined (c) presence of and (d) latency to first ‘sharp wow’ note, (e) latency to first female great call and (f) latency to male reply, and (g) total duration of singing. We also conducted a sequential analysis to compare the first 10 notes per song in the duet and predatory contexts.

The identity of each gibbon's voice was distinguishable to the experimenter, and the order in which each group member called was also noted at the time of predator presentation to facilitate analyses. Statistical analyses were conducted using SPSS software, mainly non-parametric procedures such as Mann-Whitney U-tests and Fisher's exact tests.

## Results

### Predator-induced songs

Gibbons reliably sang in response to the terrestrial, but not the raptor, predator models: clouded leopard (8/8 trials), tiger (9/9 trials), reticulated python (3/9 trials), crested serpent eagle (0/7), suggesting that singing is a firm part of these primates' natural defence to ground predators.

### Song composition: early differences

**Figure 4:**
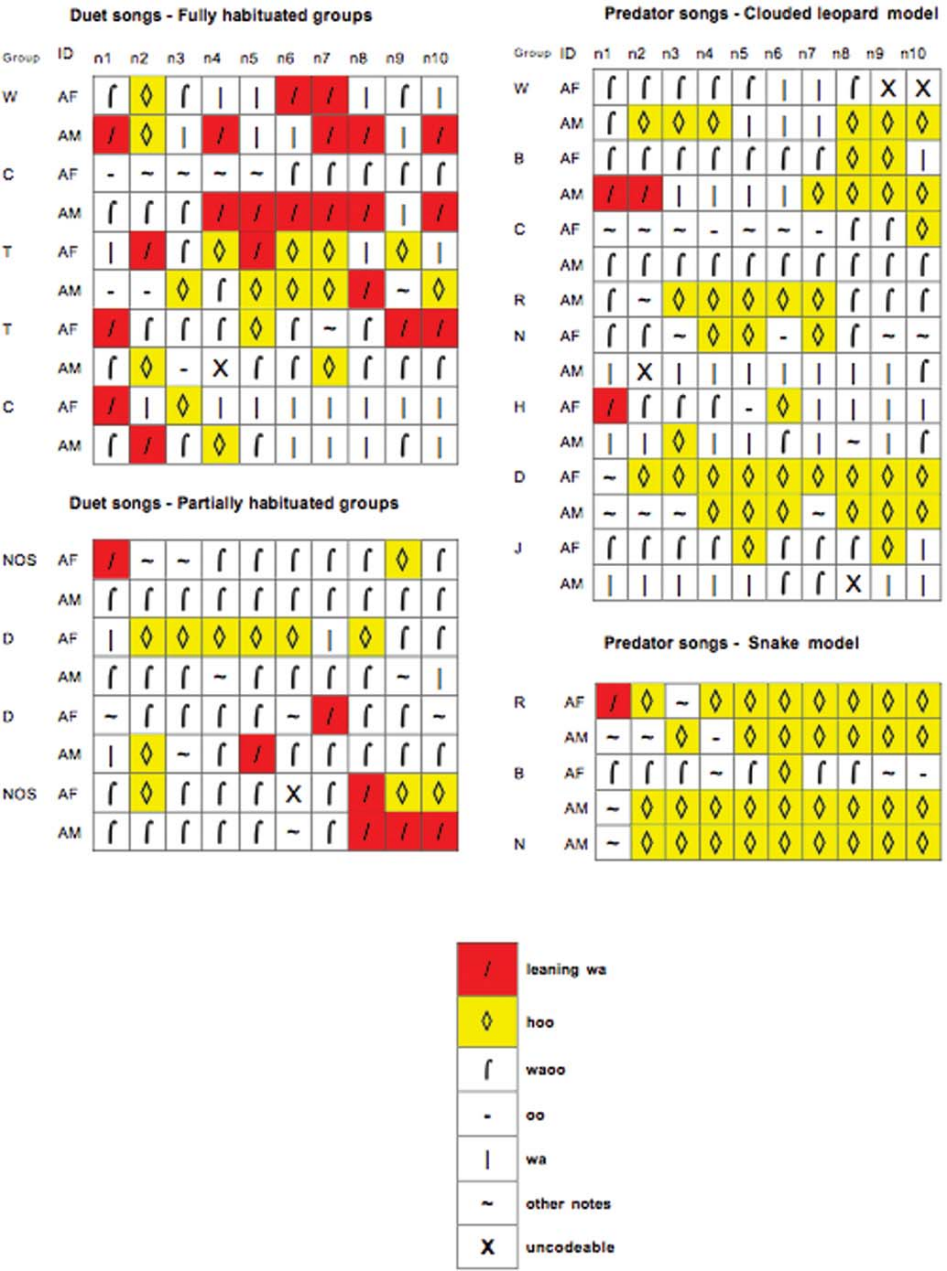
Sequential analyses of the first 10 song notes in both predatory and duet song contexts (see figure 1 for description of song notes)

Although there were no obvious acoustic differences between the songs given in duet contexts and those given in response to predators, more detailed analyses revealed a number of subtle differences. As soon as an individual began to sing (by producing loud non-‘hoo’ notes) we compared the first 10 notes for each song between the two contexts, which is roughly equivalent to about 15 s of singing (mean duration = 12.46±9.13 s, n = 38). We were particularly interested in this initial song segment because, if gibbons conveyed any information about external events, they should do so as early as possible to benefit conspecific recipients, particularly during predator encounters. Two main differences emerged. First, ‘leaning wa’ notes were significantly less likely to occur in the predatory than the duet context (Fisher's exact test, p<0.001). Second, there were significantly more ‘hoo’ notes nested within the other call units in the predatory than in the duet context (N_duet_ = 18; N_predatory_ = 20; U = 111.5; p<0.05; Mann-Whitney U-test, two-tailed). Figure 4 illustrates the patterns.

### Song composition: overall differences

Apart from the first 10 notes only, we found additional overall differences in song composition depending on context: ‘sharp wow’ notes were significantly more common in predatory than in duet songs (Fisher's Exact test, p = 0.001), appearing on average 236.4±346.8 s into a predatory song (n = 11). We also found ‘sharp wows’ in some duet songs (n = 6/14, mean latency = 71.5±47.2 s), but interestingly, they were all given by groups that had not been fully habituated to human presence (groups D, J, and NOS; table 1). It is likely, therefore, that the ‘sharp wows’ are notes given in response to any disturbance, which can be incorporated into regular duets and predator-induced songs.

Overall, songs given in the predatory context were significantly longer than songs in the duet context (mean duration = 2005.0±1560.0 s, n = 11, versus 625.9±450.7 s, n = 14, U = 28.0, p<0.01; Mann-Whitney U-test, two-tailed). Predator-induced songs were always introduced by a long series of soft ‘hoo’ notes. The number of these notes differed significantly between the predatory and the duet contexts (predatory: 100.9±110.9, n = 11; duet: 9.2±8.3, n = 14; U = 4.0, p<0.001 Mann-Whitney U-test; two-tailed). Correspondingly, the total duration of the ‘hoo’ note series in the predatory context was significantly longer than in the duet context (predatory: 158.7±290.6 s, n = 11; duet: 9.8±13.1 s, n = 14, U = 17, p = 0.001; Mann-Whitney U-test, two-tailed).

### Female great calls

**Figure 5:**
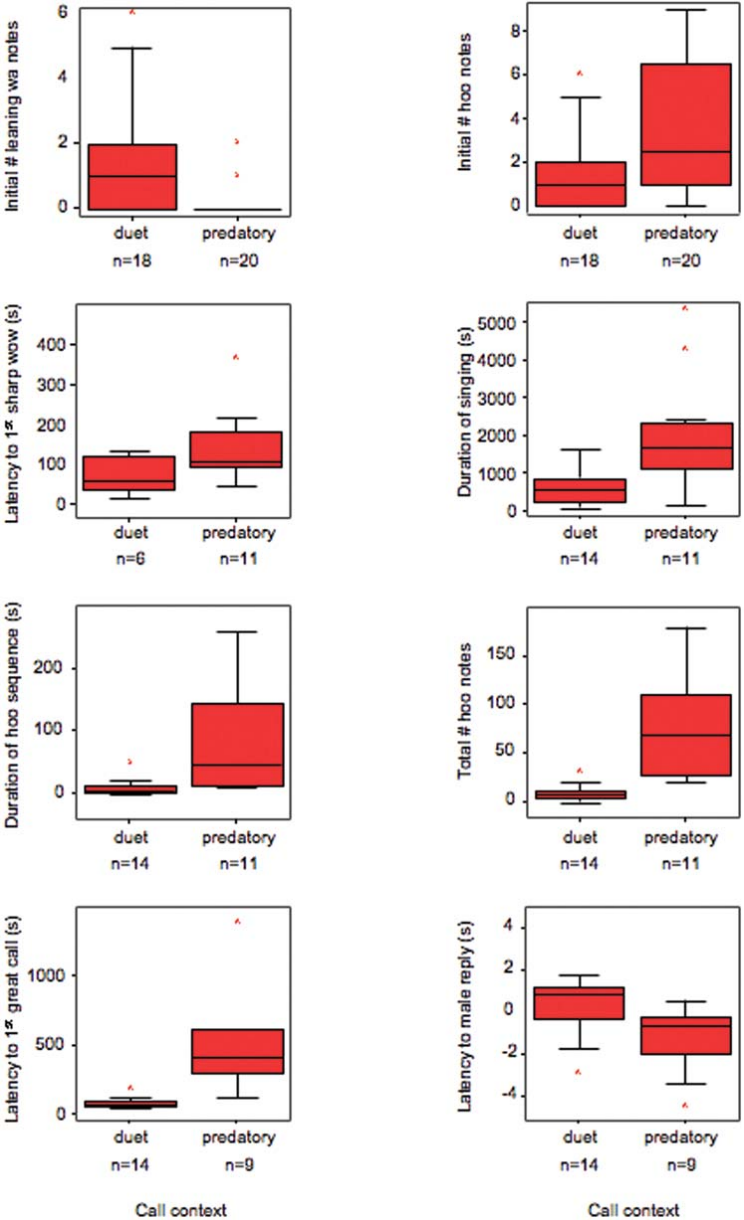
**Differences in the composition of songs given as part of normal duets or in response to predators:** The top two graphs portray the number of notes present in the sequential analyses of just the first ten song notes, where n-values represent the number of individuals (only adult males and females are included). The lower six graphs show overall compositional differences in song types according to the parameters measured. N-values represent the number of song bouts in each context.

The female great call, finally, is a stereotyped sequence of notes described as a phrase, lasting on average 17.43±1.32 s (n = 13). Females reliably produced great calls in both contexts, but during duets they were delivered significantly earlier compared to when responding to predators (duets: 80.0±35.2 s, n = 14; predatory: 682.4±669.8 s, n = 9; U = 2.0, p<0.001, Mann-Whitney U-test, two-tailed) with no overlap: Great calls during the first two minutes were reliably linked with the duet context, whereas great calls given after this time period were always associated with the presence of a predator (Fisher's exact test, p<0.001). Males usually replied to female great calls with a specific phrase, but these replies came significantly earlier in the predatory than in the duet context (predatory  = −1.3±1.7 s, n = 9; duets: 1.0±3.4 s, n = 14; U = 23.0, p = 0.012, Mann-Whitney U-test, two-tailed). Figure 5 summarises the main differences.

### Conspecific responses to gibbon songs

Sometimes, some individuals spend time away from the rest of the group. This happened on three occasions during clouded leopard model presentations (group H: adult male; group J: second adult male; group N: second adult male). In all cases, the absent individual responded with his own songs after hearing the groups' songs to the predator models, before reappearing to join the group again. We never observed this behaviour when the adult pair gave duet songs, despite the fact that in some groups the second males were often absent as well, suggesting that these individuals distinguished predator-induced from normal songs. During four other predator trials, a neighbouring group began to sing after the commencement of the study group's singing, allowing us to analyse the structure of these calls with regards to two indicators of predator-induced songs: the presence of ‘sharp wows’ and the delay of the female great call beyond 2 min. Our analyses showed that all seven response songs contained ‘sharp wow’ notes (neighbouring groups: n = 4, absent group members: n = 3). In addition, in two of the four neighbouring groups, the first female great call was delayed beyond the critical 2 min threshold, further demonstrating that these groups perceived and responded to the songs of their neighbours with the correct and matching predator songs.

We were also able to analyse a number of songs that were given in response to the regular duets by a neighbouring group (n = 4). As predicted, in all cases the first female great call was delivered during the first 2 min, indicating a normal duet context, and we never recorded any ‘sharp wow’ notes. Table 2 summarises the main results.

**Table 2:**
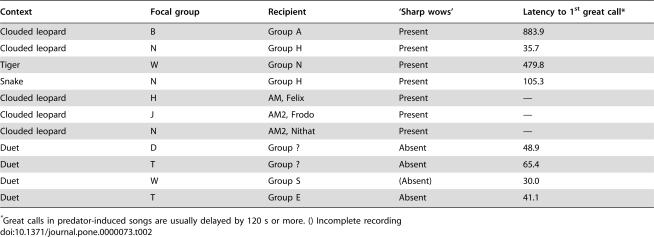
Recipients' responses to predator-induced and duet songs

Context	Focal group	Recipient	‘Sharp wows’	Latency to 1^st^ great call*
Clouded leopard	B	Group A	Present	883.9
Clouded leopard	N	Group H	Present	35.7
Tiger	W	Group N	Present	479.8
Snake	N	Group H	Present	105.3
Clouded leopard	H	AM, Felix	Present	—
Clouded leopard	J	AM2, Frodo	Present	—
Clouded leopard	N	AM2, Nithat	Present	—
Duet	D	Group ?	Absent	48.9
Duet	T	Group ?	Absent	65.4
Duet	W	Group S	(Absent)	30.0
Duet	T	Group E	Absent	41.1

* Great calls in predator-induced songs are usually delayed by 120 s or more. () Incomplete recording

## Discussion

We were interested in gibbon songs because, apart from human speech, these vocalisations provide a remarkable case of acoustic sophistication and versatility in primate communication. Individuals combine a finite number of call units into structurally more complex sequences in rule-governed ways, hereby conveying different contextual situations. Our field experiments revealed that white-handed gibbons of Khao Yai National Park, Thailand, were able to produce structurally different types of songs in the predator and duet contexts with the following differences.

First, predator-induced songs were introduced by significantly more ‘hoo’ notes than duet songs. Second, overall song duration was longer in the predator context than in the duet context. Third, the first female-specific great call was significantly delayed in a predatory song, although the acoustic structure of this phrase did not seem to differ between contexts. Fourth, males replied earlier to their own female's great calls in predation context than in duets. The absence of female great calls during the early part of a song, and hearing the male's hurried reply, in other words, are reliable indicators that the callers are singing in response to a ground predator, although this information only becomes available after a while. Fifth, predatory songs contained a smaller number of ‘leaning wa’ notes and a higher number of ‘hoo’ notes, than duet songs. The absence of ‘leaning was’ and presence of ‘hoos’ in the initial parts of a song, in other words, could function as reliable early indicators of a predator encounter. Finally, songs given to predators invariably contained ‘sharp wow’ notes, while duet songs usually did not. If ‘sharp wows’ were present in duets, then this was only in groups that were not well habituated to human observers (D, J, NOS; see Table 1), suggesting that these duet songs encoded the presence of a human observer. In all other aspects these songs were identical to normal duet songs, for example, the female great call appeared within the first 2 min and the male reply was normal, and the songs were not introduced by a series of quiet ‘hoo’ notes. We also observed that duet-based ‘sharp wows’ were given earlier in the song than the ones that were part of the predator songs, making this note a particularly interesting candidate to convey environmental events and further illustrating the remarkable flexibility underlying gibbon calling behaviour, not unlike those described for some passerine birds [32].

Gibbon songs are highly complex acoustic structures, and it may well be possible that there were other important acoustic cues present that we overlooked. For example, it appeared that male and female songs were more similar to each other in the predation than in the duet context, providing further cues that could be perceptually salient to receivers. Whatever the perceptually relevant cues, our observations also demonstrated that neighbouring groups were able to differentiate between songs given in the two contexts. In particular, we observed a predator-specific delay in the production of the first great call, as well as the inclusion of ‘sharp wow’ notes in all cases in which neighbours responded to the predator-induced song. We never observed these patterns in the response songs of neighbours to normal duets. In all observed cases, absent males began to sing while returning to the rest of the group. Again, we never observed such behaviour during normal duets. The returning males' songs always included ‘sharp wow’ notes, a convincing sign that they understood the meaning of the song produced by their group.

Why do gibbons produce these loud and conspicuous songs in response to ground predators? One function of alarm calling is to alert kin to the presence of a predator [33]. The gibbons at Khao Yai frequently change group compositions, and as a result close relatives often live in neighbouring groups [13]. For example, members of groups A and B are closely related because the adult male from B is a brother of the adult males and juvenile male from group A, perhaps explaining group A's strong response to group B's song to the clouded leopard model. Kin selection, in other words, could explain long-distance alarm calling in this and other species of gibbons [34]. In the case of large cats, empirical work has shown that primate alarm calls have a direct deterring effect on hunting behaviour [35], perhaps further explaining why it is adaptive to produce loud songs when hearing a neighbouring group singing to a predator. The quiet ‘hoo’ note series, which reliably precedes a predatory song, could function to alert immediate group members to the location of the predator, and perhaps even to signal detection to a nearby predator. Another interesting finding was that the gibbons did not produce songs to raptor models, although other types of alarm calls are produced to real raptors and models, a topic of ongoing research.

Sexual selection has been proposed as the main evolutionary mechanism for the evolution of gibbon song: males and females produce sexually dimorphic song bouts and songs are used in mate and home range defence, and in mate attraction [36]. In a recent review on the role of sexual selection on primate vocal behaviour, Snowdon [37] noted that the primate literature was weak on evidence of how individual variation in vocal behaviour impacted on reproductive decisions. In contrast, in many species of songbirds, call complexity is linked with male-male competition and mate attraction [32]. The general finding is that better quality males sing more or better than other males, usually leading to higher reproductive success because this is attractive to females or because it increases the male's ability to defend a territory. Although there is no comparable evidence for the gibbons, a number of parallels are striking. The gibbons' monogamous mating system is highly unusual, with some studies even reporting sexual polyandry [12], [27]–[28], [38]–[39]. Vocal behaviour appears to function as a powerful tool to deal with the immense sexual competition under which these primates operate, and it may not be surprising that they have evolved unusually complex vocal skills to deal with these social challenges. Not unlike humans, gibbons assemble a finite number of call units into more complex structures to convey different messages, and our data show that distant individuals are able to distinguish between different song types and infer meaning. This study thus offers first evidence of a functionally referential communication system in a free-ranging ape species, which is based on a simple phonological syntax [40].
